# Obstructive sleep apnea, cognitive impairment, and dementia: is sleep microstructure an important feature?

**DOI:** 10.1093/sleep/zsae161

**Published:** 2024-07-12

**Authors:** Nicola Andrea Marchi, Gilles Allali, Raphael Heinzer

**Affiliations:** Center for Investigation and Research in Sleep, Department of Medicine, Lausanne University Hospital and University of Lausanne, Lausanne, Switzerland; Leenaards Memory Center, Department of Clinical Neurosciences, Lausanne University Hospital and University of Lausanne, Lausanne, Switzerland; Center for Investigation and Research in Sleep, Department of Medicine, Lausanne University Hospital and University of Lausanne, Lausanne, Switzerland

Obstructive sleep apnea (OSA) and dementia are both prevalent conditions in the aging population [1, 2]. Scientific evidence indicates that OSA is associated with neurodegenerative processes [3, 4] and neurovascular damage [5, 6], which may lead to cognitive impairment and eventually dementia [7]. It has been recommended that OSA should be evaluated and treated in patients at risk for dementia [8]. However, OSA is not yet universally recognized as a modifiable risk factor for dementia [9]. Moreover, it remains unclear whether treatment of OSA can slow cognitive decline or reduce the risk of dementia [10–13]. Given the heterogeneous nature of OSA [14], it is paramount to identify which specific aspects of the disease are associated with cognitive outcomes. It has been postulated that certain key features of OSA, including intermittent hypoxemia, sleep fragmentation, and autonomic activation, may induce a cascade of pathophysiological mechanisms that ultimately result in cognitive impairment [15]. Nevertheless, previous large-scale studies on the association between OSA and cognitive impairment have primarily focused on conventional measures of breathing/hypoxemia and sleep macrostructure [16–25], while there has been a paucity of research examining sleep microstructure [26].

The current issue of *SLEEP* presents a study by Beaudin et al. that offers new insights into the relationship between OSA, sleep microstructure, and cognition [27]. The authors conducted a study on middle-aged and older adults referred to Canadian sleep centers for suspected OSA (*n* = 1142, median age 54 years, 47.2% women). The participants underwent an in-laboratory full-night or split-night polysomnography (PSG) and three cognitive tests (Montreal Cognitive Assessment, Digit-Symbol Coding Test, and Rey Auditory Verbal Learning Test). The associations between OSA, cognitive tests, and sleep microstructure metrics recorded during non-rapid eye movement (NREM) sleep (namely, sleep spindle characteristics, odds-ratio product [ORP], normalized electroencephalogram power [EEG_NP_], and delta–alpha ratio) were examined. The results showed that altered sleep spindle characteristics were associated with poorer performance on the three cognitive tests. Moreover, spindle density and EEG_NP_ were identified as mediators of the negative effect of OSA on Montreal Cognitive Assessment scores, whereas spindle power, percentage of fast spindles, and ORP were found to mediate the negative effect of OSA on Digit-Symbol Coding Test scores.

The study by Beaudin et al. is noteworthy for its large sample size of patients seen in sleep centers, which has the advantage of representing “real-life patients.” The analysis of novel sleep microstructure metrics and the mediation analysis are additional strengths of the current study. While the results of this study must be corroborated by experimental replication and longitudinal studies, they represent an advance in the identification of patients with OSA at higher risk of cognitive impairment (e.g. those with altered sleep spindle activity). Nevertheless, this study prompts the following comments. The primary analyses were performed on the first half of the full-night PSG and the diagnostic portion of the split-night PSG. This approach resulted in a low total sleep time and a near absence of rapid eye movement (REM) sleep. Although it has been suggested that a relatively low total sleep time may be sufficient to diagnose OSA [28], the possibility of misclassification of OSA severity cannot be excluded. Furthermore, the inability to measure OSA during REM sleep may have implications, as research has suggested that OSA during REM sleep may have a more deleterious effect on cognition than OSA during NREM sleep [29, 30]. Despite these limitations, it is essential to note that the authors found similar results when including only participants who underwent full-night PSG in the analyses (*n* = 619).

The study by Beaudin et al. included only three cognitive tests, which prevented a comprehensive assessment of the different cognitive functions. The most frequently observed pattern of cognitive impairment in OSA appears to be characterized by deficits in attention, working memory, and executive function [31–33]. These deficits may indicate a subcortical dysfunction, such as that seen in vascular dementia [34]. This suggests that OSA may promote microvascular damage [35]. Nevertheless, there is little research on the relationship between OSA and vascular dementia [7]. Conversely, studies have indicated that OSA may be associated with an increased risk of Alzheimer’s disease [7, 36]. However, to the best of our knowledge, there is limited evidence that OSA is associated with an amnestic syndrome [37], which is the typical presentation of Alzheimer’s disease. Future research should examine whether the reduced episodic memory observed in OSA [37] reflects an impairment in retrieval strategies (which could suggest a frontal lobe-dependent executive dysfunction) or a deficit in consolidation/storage (which suggests an amnestic syndrome of the hippocampal type). These future studies are crucial in the current era of disease-modifying therapies for Alzheimer’s disease [38, 39], as an extensive population screening is mandatory to identify the underlying causes of memory decline in aging.

While beyond the scope of the study by Beaudin et al., it is important to note that the association between OSA and cognitive impairment may be moderated by age and sex. However, previous studies examining these moderating effects have yielded inconclusive results [19, 23, 40–42]. In contrast, a moderating effect of the apolipoprotein E4 (ApoE4) allele has been observed in a range of population-based cohorts [16, 18, 19, 23]. These studies have consistently observed a stronger association between OSA and cognitive dysfunction in ApoE4 carriers compared with ApoE4 non-carriers [16, 18, 19, 23]. Some comorbidities, such as obesity [19] and hypertension [43], have also been proposed to moderate the association between OSA and cognition; however, further research is needed to substantiate these observations.

In OSA, respiratory events lead to activation of the autonomic nervous system (ANS), which can be detected by variations in the heart rate (on the electrocardiogram signal) [44], as well as in the pulse rate [45, 46] and the pulse wave amplitude (on the pulse oximeter signal) [47–49]. Only a few investigations have been conducted on PSG markers of the ANS, yet these have been found to be associated with cognitive impairment [44] and the incidence of dementia [46] among patients with OSA. Therefore, PSG markers of the ANS should be accorded greater consideration in future research.

**Figure 1.**
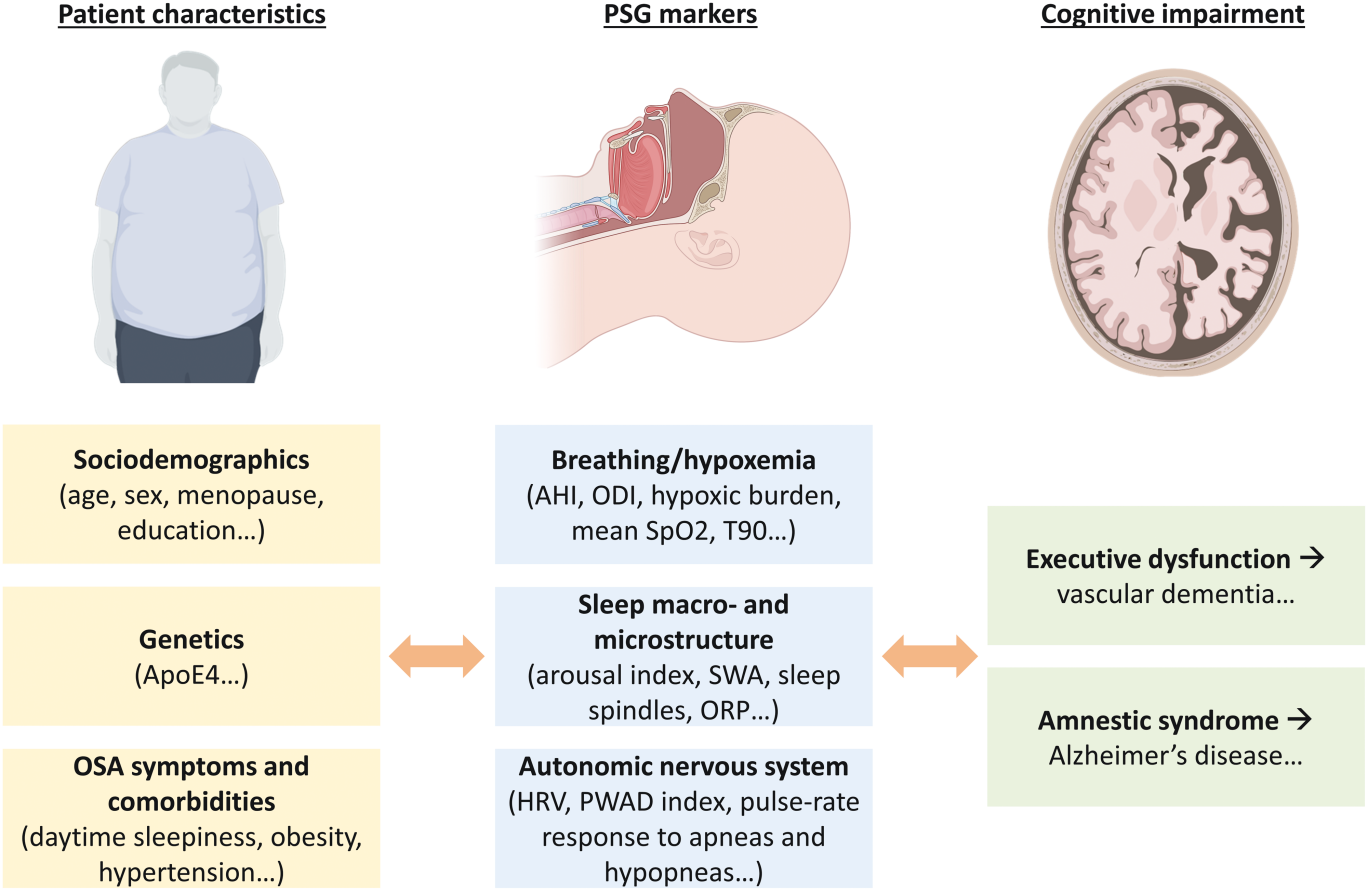
Conceptual framework for studying the relationship between OSA and cognitive impairment. The figure presents a list of factors that could be subjected to investigation in order to elucidate the relationship between OSA and cognitive impairment. It should be noted that this list is provided for illustrative purposes only and is not intended to be exhaustive. The images were adapted from BioRender.com. Abbreviations: AHI, apnea–hypopnea index; ApoE4, apolipoprotein E4; HRV, heart rate variability; ODI, oxygen desaturation index; OSA, obstructive sleep apnea; ORP, odds-ratio product; PSG, polysomnography; PWAD, pulse wave amplitude drop; SpO2, oxygen saturation; SWA, slow-wave activity; T90, sleep time with oxygen saturation <90%.

In conclusion, the study by Beaudin et al. represents a valuable contribution to this field of research. Nevertheless, it is crucial to acknowledge that sleep microstructure represents only one aspect of the intricate relationship between OSA and cognition. Future research should consider a set of factors, including patient characteristics, PSG markers that reflect the diverse pathophysiological mechanisms of OSA, and the pattern of cognitive deficits (Figure 1). This framework has the potential to facilitate the identification of OSA phenotypes associated with cognitive impairment. The identification of such OSA phenotypes will also be critical in selecting patients for new interventional studies on the effect of OSA treatment on cognition. Given that it has been estimated that 40% of dementia cases could be postponed or prevented by addressing modifiable risk factors [9], demonstrating that treatment of OSA can slow cognitive decline or reduce the risk of dementia could have a profound impact on public health.
